# A Comparison of Different Modeling Techniques in Predicting Mortality With the Tilburg Frailty Indicator: Longitudinal Study

**DOI:** 10.2196/31480

**Published:** 2022-03-30

**Authors:** Tjeerd van der Ploeg, Robbert Gobbens

**Affiliations:** 1 Faculty of Health, Sports and Social Work Inholland University of Applied Sciences Amsterdam Netherlands; 2 Zonnehuisgroep Amstelland Amstelveen Netherlands; 3 Department Family Medicine and Population Health Faculty of Medicine and Health Sciences University of Antwerp Antwerp Belgium

**Keywords:** modeling techniques, area under the receiver operating characteristic curve, bootstrapping, validation, predictor variable importance

## Abstract

**Background:**

Modern modeling techniques may potentially provide more accurate predictions of dichotomous outcomes than classical techniques.

**Objective:**

In this study, we aimed to examine the predictive performance of eight modeling techniques to predict mortality by frailty.

**Methods:**

We performed a longitudinal study with a 7-year follow-up. The sample consisted of 479 Dutch community-dwelling people, aged 75 years and older. Frailty was assessed with the Tilburg Frailty Indicator (TFI), a self-report questionnaire. This questionnaire consists of eight physical, four psychological, and three social frailty components. The municipality of Roosendaal, a city in the Netherlands, provided the mortality dates. We compared modeling techniques, such as support vector machine (SVM), neural network (NN), random forest, and least absolute shrinkage and selection operator, as well as classical techniques, such as logistic regression, two Bayesian networks, and recursive partitioning (RP). The area under the receiver operating characteristic curve (AUROC) indicated the performance of the models. The models were validated using bootstrapping.

**Results:**

We found that the NN model had the best validated performance (AUROC=0.812), followed by the SVM model (AUROC=0.705). The other models had validated AUROC values below 0.700. The RP model had the lowest validated AUROC (0.605). The NN model had the highest optimism (0.156). The predictor variable “difficulty in walking” was important for all models.

**Conclusions:**

Because of the high optimism of the NN model, we prefer the SVM model for predicting mortality among community-dwelling older people using the TFI, with the addition of “gender” and “age” variables. External validation is a necessary step before applying the prediction models in a new setting.

## Introduction

Predicting the survival probability of patients is important for various purposes in biomedaical research, such as patient counseling, medical decision-making, and benchmarking. The traditional analysis of survival problems uses Kaplan-Meier analysis and Cox regression modeling to predict the survival probability depending on various predictor variables.

Prediction is complicated by the specification of the model structure, such as the inclusion of main effects, potential nonlinearities, and statistical interaction [1-3]. While most prediction models for binary endpoints are still based on logistic regression (LR) analysis, there is increasing interest in other, more modern techniques, such as neural networks (NNs), random forests (RFs), and support vector machines (SVMs). These techniques hold the promise of better capturing nonlinearities and interactions in medical data and are, therefore, attractive in possibly providing better predictions [4].

NNs were used in 1998 for the analysis of survival data [5], and in 2007, applications of random survival forests were described [6]. SVMs were used in the context of breast cancer survival and chemotherapy [7]. In 2009, prognostic indexes were compared using modern techniques and Cox regression analysis in breast cancer data [8].

The aim of this study was to determine the best modeling technique for the prediction of mortality in a sample of community-dwelling older people by components of frailty using a follow-up period of 7 years. Frailty is the focus of much attention in practice, policy, and research. This is hardly surprising, since frailty in older people is predictive for disability [9], an increase in health care use [10], lower quality of life, and mortality [11].

Frailty is often operationalized by physical components, for example, in the phenotype of frailty by Fried et al [9]. However, only paying attention to physical limitations that older people may have or experience can lead to fragmentation of care [12] and then, potentially, to a reduction of quality of care and a decrease in quality of life of older people. Therefore, we used the Tilburg Frailty Indicator (TFI), a multidimensional scale including physical, psychological, and social components, for assessing frailty [13]. The TFI was developed on the basis of an extensive literature review and consultation with experts [12-14] and has shown good psychometric properties [15].

Five studies have examined the predictive value of the TFI for mortality [16-20]. Only one of these previous studies used the original TFI and conducted the study among community-dwelling older people [20]. In this Dutch cohort study with 2-year follow-up including 2420 community-dwelling older people, the area under the receiver operating characteristic curve (AUROC) for predicting mortality using the TFI was 0.620 [20]. Previous studies that compared alternative modeling techniques for predicting survival made use of pseudovalues [21,22]. In this study, we focused on 7-year mortality.

## Methods

### Study Population and Data Collection

In June 2008, the TFI was sent to a sample of 1154 community-dwelling older people aged 75 years and older randomly drawn from the register of the municipality in Roosendaal, a town of 78,000 inhabitants in the Netherlands. A total of 484 participants completed the questionnaire (41.94% response rate), which, complementary to the TFI, also contained measures for assessing quality of life and disability [23,24]. As in a previous study, the data from 5 participants were left out of the analyses as they had too many omissions, leaving a data set of 479 participants [23].

### Measures

#### Frailty

The TFI contained 15 components of frailty distributed over physical, psychological, and social frailty. The components of physical frailty included the following: physically unhealthy, unexplained weight loss, difficulty in walking, difficulty in maintaining balance, poor hearing, poor vision, lack of strength in the hands, and physical tiredness. Psychological frailty consisted of problems with memory, feeling down, feeling nervous or anxious, and being unable to cope with problems. Social frailty included living alone, lack of social relations, and lack of social support. For the exact content and the scoring of the TFI, we refer to a previous study [13].

#### Mortality

In August 2015, the municipality of Roosendaal provided the mortality dates of the participants who completed the questionnaire in 2008. With these dates, 7-year mortality was defined.

### Data and Data Imputation

For the modeling, we used the data set (N=479) with the 15 frailty components, gender (“male” or “female”), and the dichotomous transformed age variable (“≤80” or “>80” years) as predictor variables and 7-year mortality (“alive” or “dead”) as the outcome variable. We imputed data for the missing values using the MICE (Multivariate Imputation by Chained Equations) package (m=5 and methods=“logreg”) in R software (version 3.4.4; The R Foundation) [25]. The first imputed data set was used for the modeling.

### Modeling Techniques

#### Overview

We compared eight modeling techniques to predict 7-year mortality: (1) LR, (2) least absolute shrinkage and selection operator (LASSO), (3) SVM, (4) NN, (5) recursive partitioning (RP), (6) RF, (7) hill-climbing (HC) Bayesian network, and (8) naïve Bayes (NB) network.

Here, we list the main characteristics of the evaluated modeling techniques, based on the work of several authors [2,3,26-30] and an earlier publication of the first author [31].

#### Logistic Regression

LR is a type of regression analysis that is often used in medical research to model the probability of a dichotomous endpoint using a linear function of the predictors. Predictor variables may be either continuous or categorical. LR uses a logistic transformation to calculate the probability of a dichotomous outcome. Regression coefficients were estimated by maximum likelihood [31].

#### Least Absolute Shrinkage and Selection Operator

LASSO is quite similar to linear regression and LR, but it adds a penalty for nonzero regression coefficients using the sum of their absolute values. As a result, small regression coefficients are set to zero. Regression coefficients were estimated by maximum likelihood [31].

#### Support Vector Machine

An SVM performs classification tasks by constructing hyperplanes with a margin in a multidimensional space that separates cases from different classes. An SVM can perform a nonlinear classification or regression task using different kernels (ie, radial, linear, and polynomial). The tuning parameters for SVMs are the C parameter (cost), which regulates the margin width, and the gamma parameter for the kernel calculation. SVM claims to be a robust classification and regression technique that maximizes the predictive accuracy of a model without overfitting the training data. SVM may be particularly suited to analyze data with large numbers of predictor variables [31].

#### Neural Network

An NN simulates a large number of interconnected simple processing units that are arranged in layers. There are three parts in an NN: an input layer, with units representing the predictor variables; one or more hidden layers; and an output layer, with a unit representing the endpoint. The units are connected with varying connection strengths or weights. Input data are presented to the input layer, and values are propagated from there to the next layer. Then, a prediction is delivered from the output layer. The NN learns by examining individual records, generating a prediction for each record and making adjustments to the weights whenever it makes an incorrect prediction. The adjustments are based on the gradient descent algorithm to minimize the prediction error. This process is repeated many times, and the NN continues to improve its predictions until the magnitude of the gradient is less than a certain threshold (eg, 0.00005). Once trained, the NN can be applied to new records for which the endpoint is unknown. The crucial parameters of an NN are the size parameter (ie, number of units in the layer) and the decay parameter, which penalizes large weights in the model to avoid overfitting [31].

#### Recursive Partitioning

RP is a modeling technique that uses RP to split the training records into segments with similar endpoint values. The modeling starts by examining the input variables to find the best split, measured by the reduction in an impurity index that results from the split. The split defines two subgroups, each of which is subsequently split into two further subgroups and so on, until a stopping criterion is met. The commonly used parameter for RP is the cp parameter (cost complexity factor). A cp value of 0.001, for example, regulates that a split must decrease the overall lack of fit by a factor of 0.001 [31].

#### Random Forest

RF is an ensemble classifier that consists of many decision trees. In case of classification, RF outputs the class that is the mode among the classes from individual trees. In case of regression, RF outputs the value that is the mean of the values output from individual trees. Each tree is constructed using a bootstrap sample from the original data. A tree is grown by recursively partitioning the bootstrap sample based on optimization of a split rule. In regression problems, the split rule is based on minimizing the mean squared error, whereas in classification problems, the Gini index is commonly used. At each split, a subset of candidate variables are tested for the split rule optimization, similar to RP modeling. For prediction, a new sample is pushed down the tree. This procedure is iterated over all trees in the ensemble. Key parameters are the number of trees and the number of candidate variables [31].

#### Hill-Climbing Bayesian Network

A Bayesian network is a mathematical construct that compactly represents a joint probability distribution among a set of variables. Bayesian networks are frequently employed for modeling domain knowledge in decision support systems, particularly in medicine. Learning Bayesian networks is connected with variable selection for classification and has been used to design algorithms that optimally solve the problem under certain conditions. The HC Bayesian network is a score-based search algorithm to learn a Bayesian network structure with a sparse set of variables [32].

#### Naïve Bayes Network

The NB model is technically a special case of a Bayesian network. The NB model assumes that all the features are conditionally independent of each other and that, therefore, the Bayesian rule for probability can be applied. Usually this independence assumption works well for most cases, even if in actuality they are not really independent [32].

### Analysis

For all analyses, we used R (version 3.4.4; The R Foundation) [33].

#### Statistics

We used counts and percentages to describe the baseline characteristics of the participants. The chi-square test was used to compare dichotomous variables. A *P* value of less than .05 was considered significant. Cramer V, a statistic derived from the chi-square value, was used as an association measure: values toward zero indicate weak association and values toward 1 indicate strong association. The predictive performance of the models was measured using the AUROC. An AUROC greater than 0.700 was considered as an indication of good predictive performance [3].

#### Relative Importance of the Predictor Variables

The relative importance of a predictor variable in a model was calculated using the Permutation Feature Importance algorithm with 1000 repetitions [34,35]. We used the decrease in median apparent AUROC as the measure for ranking the relative importance of a predictor variable.

#### Bootstrap Validation of the Models

Each model was validated using the bootstrap validation procedure as proposed by Efron and Tibshirani [36]. Here, we describe the bootstrap validation procedure. First, a model was developed on the original data set, and the AUROC of that model for the original data set was calculated (ie the apparent AUROC). Then, a sample with replacement was drawn from the original data set with a size equal to the size of the original data set. This sample was called the bootstrap sample. For this bootstrap sample, the model was developed again, and the AUROC for that bootstrap sample was calculated (ie, the developed AUROC). This model was then applied to the original data set and the AUROC was calculated (ie, the validated AUROC). The difference between the developed AUROC and the validated AUROC is defined as the optimism of the model. By subtracting this optimism from the apparent AUROC, we obtain the corrected AUROC. This process was repeated 100 times.

### Ethics Approval and Consent to Participate

All procedures performed in studies involving human participants were in accordance with the ethical standards of the institutional and national research committee, and with the 1964 Declaration of Helsinki and its later amendments or comparable ethical standards. For this study, medical ethics approval was not necessary because particular treatments or interventions were not offered or withheld from respondents. Moreover, the integrity of the respondents was not encroached upon as a consequence of participating in this study, which is the main criterion in medical-ethical procedures in the Netherlands [37]. Informed consent related to details of the study and maintaining confidentiality was observed.

## Results

### Participant Characteristics and Variable Association

Table 1 presents the descriptive statistics and the univariate *P* values of the chi-square test for the participants at baseline in relation to 7-year mortality. Five predictor variables (ie, gender, poor hearing, poor vision, feeling down, and living alone) showed univariate *P* values equal to or greater than .05. Three of these predictor variables (ie, poor hearing, poor vision, and living alone) had *P* values equal to or greater than .20.

**Table 1:** Participant characteristics.

Characteristic (category)	Alive (n=317), n (%)	Dead (n=162), n (%)	*P* value^a^
Gender (male)	130 (41.0)	77 (47.5)	.17
Age (>80 years)	119 (37.5)	85 (52.5)	.002
Physically unhealthy (yes)	71 (22.4)	70 (43.2)	<.001
Unexplained weight loss (yes)	15 (4.7)	21 (13.0)	.001
Difficulty in walking (yes)	121 (38.2)	110 (67.9)	<.001
Difficulty in maintaining balance (yes)	86 (27.1)	84 (51.9)	<.001
Poor hearing (yes)	110 (34.7)	65 (40.1)	.24
Poor vision (yes)	65 (20.5)	38 (23.5)	.46
Lack of strength in the hands (yes)	96 (30.3)	68 (42.0)	.01
Physical tiredness (yes)	120 (37.9)	98 (60.5)	<.001
Problems with memory (yes)	21 (6.6)	25 (15.4)	.002
Feeling down (yes)	121 (38.2)	72 (44.4)	.19
Feeling nervous or anxious (yes)	87 (27.4)	61 (37.7)	.02
Unable to cope with problems (yes)	42 (13.2)	34 (21.0)	.03
Living alone (yes)	154 (48.6)	75 (46.3)	.64
Lack of social relations (yes)	174 (54.9)	108 (66.7)	.01
Lack of social support (yes)	44 (13.9)	34 (21.0)	.046

^a^Univariate *P* values were based on the chi-square test for the participants at baseline in relation to 7-year mortality.

**Figure 1:**
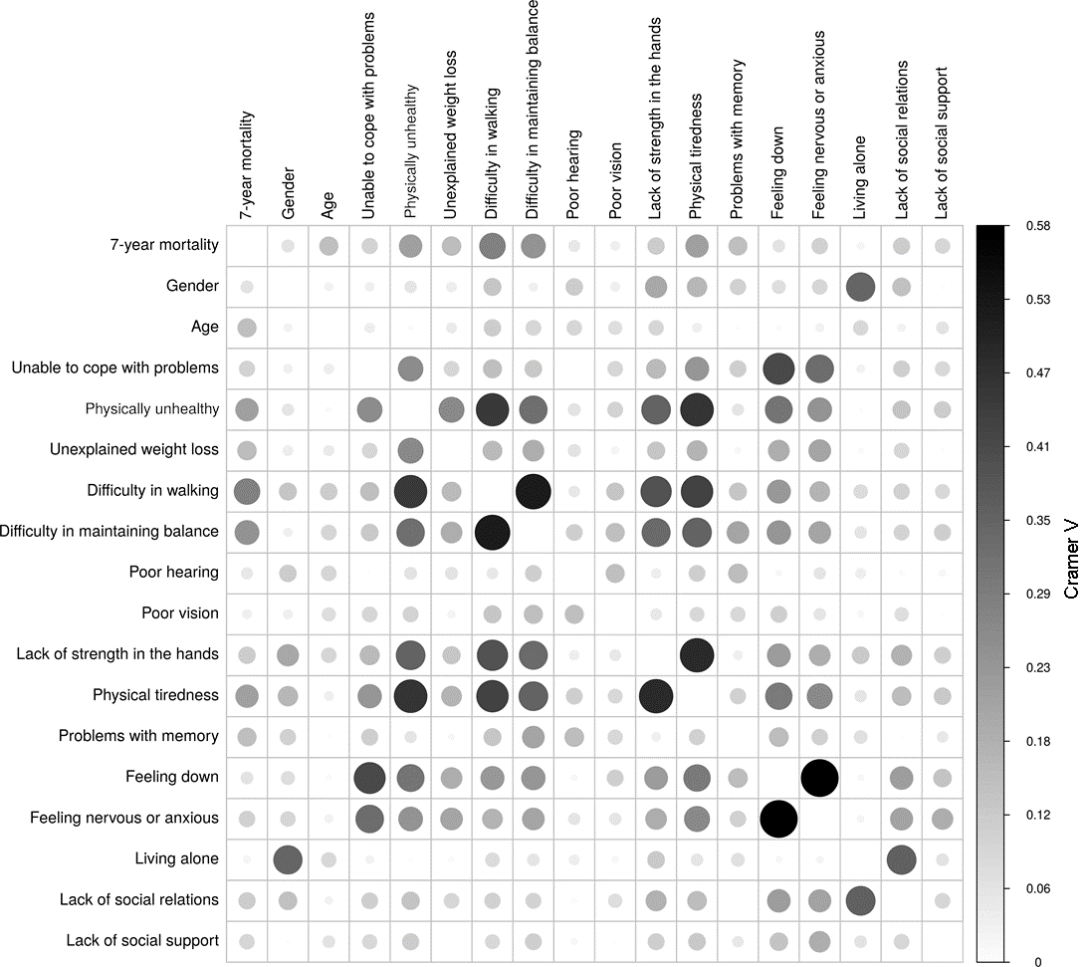
Strength of the associations between the predictor variables (darker colour indicates stronger association).

A priori, we could assume that the predictor variables listed in Table 1 have no association. Figure 1 visualizes the association of the predictor variables with each other and with the outcome variable based on Cramer V, as described in the Statistics section. For example, there are strong associations between “difficulty in walking” and “difficulty in maintaining balance” and between “feeling anxious or nervous” and “feeling down.”

### Prediction of 7-Year Mortality by the 15 Frailty Components, Gender, and Age

We applied each modeling technique, as mentioned in the Modeling Techniques section, to the data set mentioned in the Measures section and validated the models with bootstrapping (100 repetitions) as described in the Analysis section. Table 2 presents the performance characteristics of the models. The corrected AUROC values varied from 0.605 for RP to 0.812 for NN. The optimism of the NN model was high (0.156). The optimism of the RF model showed a 95% CI containing a value of zero, indicating that the RF model was not overfitted. However, the performance of the RF model was low (apparent AUROC=0.665).

**Table 2:** Performance characteristics of the models.

Model	Apparent AUROC^a,b^	Developed AUROC^c^, mean (95% CI)	Validated AUROC^d^, mean (95% CI)	Optimism^e^, mean (95% CI)		Corrected AUROC^f^
Logistic regression	0.743	0.765 (0.723 to 0.804)	0.721 (0.694 to 0.735)	0.045 (0.006 to 0.084)	0.698	
LASSO^g^	0.742	0.762 (0.717 to 0.799)	0.720 (0.700 to 0.733)	0.043 (0.006 to 0.084)	0.699	
SVM^h^	0.764	0.804 (0.771 to 0.837)	0.745 (0.729 to 0.763)	0.059 (0.020 to 0.089)	0.705	
Neural network	0.967	0.989 (0.974 to 0.998)	0.834 (0.793 to 0.868)	0.156 (0.123 to 0.197)	0.812	
Recursive partitioning	0.680	0.771 (0.711 to 0.826)	0.696 (0.643 to 0.731)	0.075 (0.034 to 0.116)	0.605	
Random forest	0.665	0.867 (0.835 to 0.899)	0.873 (0.851 to 0.898)	–0.007 (–0.056 to 0.042)	0.671	
HC^i^ Bayesian network	0.649	0.674 (0.522 to 0.738)	0.654 (0.521 to 0.689)	0.020 (–0.009 to 0.061)	0.629	
Naïve Bayes	0.704	0.717 (0.683 to 0.759)	0.704 (0.704 to 0.704)	0.014 (–0.021 to 0.055)	0.690	

^a^AUROC: area under the receiver operating characteristic curve.

^b^The apparent AUROC is the AUROC of the model for the original data set.

^c^The developed AUROC is the AUROC of the redeveloped model on the bootstrap sample.

^d^The validated AUROC is the AUROC of the validated model.

^e^The model optimism is the difference between the developed AUROC and the validated AUROC.

^f^The corrected AUROC is the AUROC obtained by subtracting the optimism from the apparent AUROC.

^g^LASSO: least absolute shrinkage and selection operator.

^h^SVM: support vector machine.

^i^HC: hill-climbing.

### Relative Importance of the Predictor Variables for the NN Model and the SVM Model

**Figure 2:**
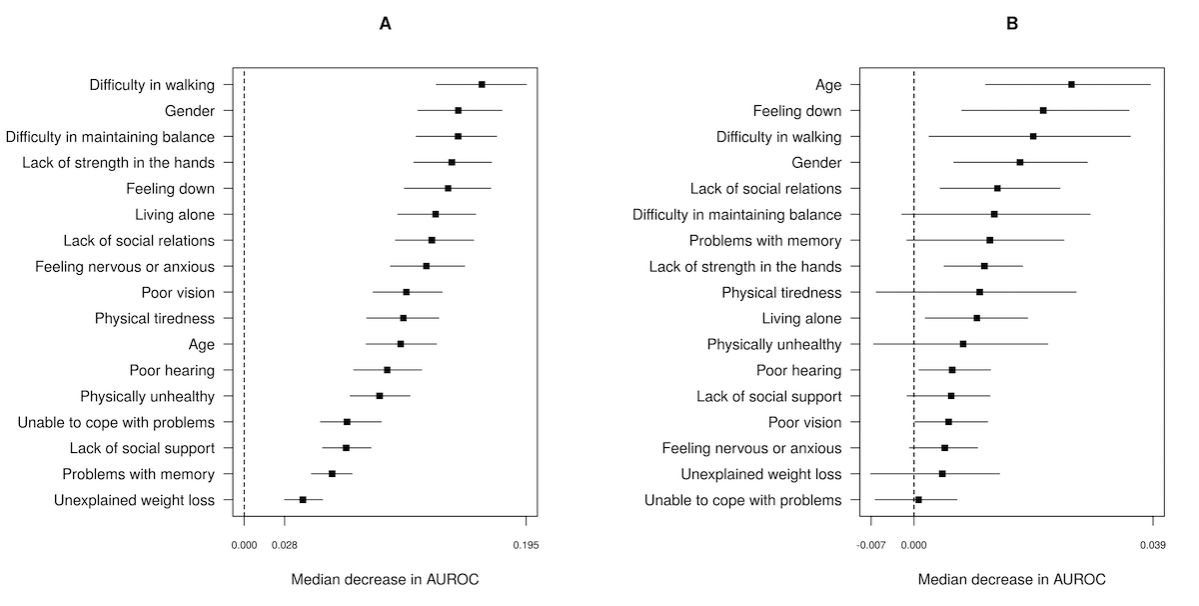
Median decrease in apparent AUROC and 95% CI (whiskers) for the neural network model (A) and the support vector machine model (B). AUROC: area under the receiver operating characteristic curve.

The NN model and the SVM model had corrected AUROCs above 0.700, indicating a good performance. Figure 2 shows the relative importance of the predictor variables for these models, calculated as described in the Analysis section. The depicted points correspond to the median decrease in apparent AUROC, and the boundaries of the bands illustrate the 95% CI for the decrease in apparent AUROC. The dashed line corresponds to a value of zero. If the 95% CI contains the value of zero, the predictor variable has no significant importance for the model. The predictor variables “difficulty in walking,” “gender,” and “difficulty in maintaining balance” had the highest relative importance in the NN model; the predictor variables “age,” “feeling down,” and “difficulty in walking” had the highest relative importance in the SVM model. For the relative importance of the predictor variables in the other models, we refer to Figures S1-S3 in Multimedia Appendix 1.

## Discussion

### Principal Findings

Many studies have observed that frailty is associated with mortality among community-dwelling older people [38]. To date, only one study used the original version of the TFI for the prediction of mortality among Dutch community-dwelling older people, using a 2-year follow-up [20].

The aim of this study was to determine the best modeling technique for predicting mortality in a Dutch sample of 479 community-dwelling older people with a 7-year follow-up by assessing frailty with the TFI. We compared eight modeling techniques to develop prediction models. The classical approach for developing a prediction model for a dichotomous outcome is to use the LR technique or the penalized version, LASSO. Both techniques are based on a linear combination of the predictor variables (see Modeling Techniques section). The other evaluated techniques are able to capture nonlinearity and can deal with interaction of the predictor variables [39].

Of the 15 components of the TFI, three had *P* values equal to or greater than .20 (ie, poor hearing, poor vision, and living alone); normally, these variables would not be included in a multivariate analysis. However, removing these components from the TFI on the basis of this study is not recommended. The inclusion of sensory difficulties in a screening instrument such as the TFI has major consequences in terms of the prevalence and prediction of adverse outcomes (eg, hospitalization) [40]. Therefore, for all techniques, we used all 15 components of the TFI; we also added “gender” and “age” as predictor variables.

The simplest way to construct a prediction model is to calculate the sum score of the TFI components, adding 1 if the participant is “male” and adding 1 again if the participant is “>80 years.” Therefore, the maximum sum score is 17. The apparent AUROC for this sum score model in predicting mortality was 0.680. The algorithm of the LR modeling technique led to a model with an apparent AUROC of 0.743 in predicting mortality. The LASSO model had an apparent AUROC of 0.742, with only the following predictors: “age,” “physically unhealthy,” “difficulty in walking,” “difficulty in maintaining balance,” and “physical tiredness.” These results show that applying algorithms paid off above using just the simple approach.

LR and LASSO are regression-based techniques. An SVM is a modern, advanced modeling technique that is able to discriminate between the categories “alive” and “dead” using high-dimensional hyperplanes to separate them. The corrected AUROC of the SVM model was 0.705 and the optimism was 0.059.

The NN model showed the highest apparent and corrected AUROCs. However, the optimism of the NN model was 0.156. This and the fact that an NN model has a black box character makes the application of an NN model unattractive in predicting mortality in our study.

We calculated the relative importance of the predictors in the NN model as well as in the SVM model. It is obvious that the top three important variables differed for both models. However, the predictor variable “difficulty in walking” was present in the top three of both models. This was also the case with the other six models. In general, each model has its own ranking of important variables due to the underlying algorithm [21].

Models provided by the RP modeling technique are considered attractive in a medical setting because they show a decision tree. In our study, the RP model performed poorly (corrected AUROC=0.605). The RF modeling technique is attractive because it claims to provide models without overfitting [26]. This is in line with our study because the 95% CI for bootstrap validation for the optimism was –0.056 to 0.042, indicating that the optimism does not differ significantly from zero. The performance of the RF model was also somewhat poor (corrected AUROC=0.671). However, the RF modeling technique is considered as an obvious improvement over the RP modeling technique [41,42]. It is, hence, remarkable that the RP modeling technique has, until recently, been advocated for as the preferred modeling technique for prediction in some disease areas, such as trauma [4].

Bayesian networks, with their underlying algorithms, are especially suited for capturing and reasoning with uncertainty. They have been applied in biomedicine and health care for more than a decade now and are still gaining in popularity. Bayesian networks are used in clinical epidemiology for the construction of disease prediction models and within bioinformatics for the interpretation of microarray gene expression data, for instance [43]. In our study, we evaluated two Bayesian network algorithms, HC Bayesian network and NB, for the prediction of 7-year mortality. The HC Bayesian network and NB algorithms showed corrected AUROCs of 0.629 and 0.690, respectively. The NB algorithm used all predictor variables, whereas the HC Bayesian network algorithm was developed to determine a sparse set of predictor variables. For our data set, the HC Bayesian network algorithm only used the predictor variable “difficulty in walking” for the prediction of 7-year mortality.

The internal validation of the models was done using bootstrapping with 100 repetitions to get insight into the amount of optimism. Other examples of internal validation techniques are split-sample and cross-validation techniques [44]. While the interest in the development, validation, and clinical application of prediction models is increasing, a recent systematic review showed that only a quarter of the studies reported prediction models with internal as well as external validation [45,46]. External validation aims to address the performance of a prediction model in a different but plausibly related data set, which still represents the underlying domain. This validation step is widely considered necessary before implementing a developed prediction model in practice [47,48]. We support this notion, and we strongly suggest validating the developed models in our study in the data sets that were used in other studies [16-20].

A number of limitations of this study should be addressed. First, our sample consisted exclusively of people living independently in the municipality of Roosendaal. Therefore, the generalizability of the findings can be questioned. Second, the TFI is a frailty instrument using self-reported data, so frailty is subjectively assessed. However, the construct validity of the TFI has been determined in detail using objective measurements [13]. Third, we used default settings for the modeling techniques. This holds for LR and LASSO as well as for the modern methods where various specific parameters might be fine-tuned to the development setting [1,3,42]. Further tuning of parameters to specific issues in a particular development data set might obviously improve the apparent performance, but we doubt that substantial improvement would be achieved in the validated external performance.

### Conclusions

In conclusion, this study has shown that the NN and SVM models outperformed the other six models (corrected AUROCs>0.700). Because of the high optimism of the NN model, we prefer the SVM model for predicting mortality among community-dwelling older people using the 15 components of the TFI, with the addition of “gender” and “age.” Furthermore, external validation is a necessary step before applying the prediction models in a new setting.

## Appendix

Multimedia Appendix 1 Relative importance of the predictor variables for the models.

## Abbreviations

AUROC: area under the receiver operating characteristic curve
HC: hill-climbing
LASSO: least absolute shrinkage and selection operator
LR: logistic regression
MICE: Multivariate Imputation by Chained Equations
NB: naïve Bayes
NN: neural network
RF: random forest
RP: recursive partitioning
SVM: support vector machine
TFI: Tilburg Frailty Indicator

## Data Availability

The data set used and analyzed during this study is available from the corresponding author on reasonable request.
