# Metadata Correction: A Comparison of Different Modeling Techniques in Predicting Mortality With the Tilburg Frailty Indicator: Longitudinal Study

**DOI:** 10.2196/31479

**Published:** 2022-04-08

**Authors:** Tjeerd van der Ploeg, Robbert Gobbens

**Affiliations:** 1 Faculty of Health, Sports and Social Work Inholland University of Applied Sciences Amsterdam Netherlands; 2 Zonnehuisgroep Amstelland Amstelveen Netherlands; 3 Department Family Medicine and Population Health Faculty of Medicine and Health Sciences University of Antwerp Antwerp Belgium

In “A Comparison of Different Modeling Techniques in Predicting Mortality With the Tilburg Frailty Indicator: Longitudinal Study” (JMIR Med Inform 2022;10(3):e31480) the authors noted the following two errors.

1. In the originally published article, author affiliations appeared as follows:

Tjeerd van der Ploeg, PhD; Robbert Gobbens, PhD

Faculty of Engineering, Design and Computer Technology, Inholland University of Applied Sciences, Alkmaar, Netherlands

The corrected affiliations for the authors are as follows:

Tjeerd van der Ploeg^1^, PhD; Robbert Gobbens^1,2,3^, PhD

^1^Faculty of Health, Sports and Social Work, Inholland University of Applied Sciences, Amsterdam, Netherlands

^2^Zonnehuisgroep Amstelland, Amstelveen, Netherlands

^3^3Department Family Medicine and Population Health, Faculty of Medicine and Health Sciences, University of Antwerp, Antwerp, Belgium

2. In the originally published article, the corresponding author's address appeared as follows:

Tjeerd van der Ploeg, PhD

Faculty of Engineering, Design and Computer Technology

Inholland University of Applied Sciences

Bergerweg 200

Alkmaar, 1817 MR

Netherlands

The address has been corrected as follows:

Tjeerd van der Ploeg, PhD

Faculty of Health, Sports and Social Work

Inholland University of Applied Sciences

De Boelelaan 1109

Amsterdam, 1081 HV

Netherlands

The correction will appear in the online version of the paper on the JMIR Publications website on April 8, 2022, together with the publication of this correction notice. Because this was made after submission to PubMed, PubMed Central, and other full-text repositories, the corrected article has also been resubmitted to those repositories.

