# Supplementary material for: A Comparison of Different Modeling Techniques in Predicting Mortality With the Tilburg Frailty Indicator: Longitudinal Study
Source: JMIR Med Inform. 2022 Mar 30;10(3):e31480. doi: 10.2196/31480 (PMC8992962; doi:10.2196/31480)
Supplement: Multimedia Appendix 1 [file medinform_v10i3e31480_app1.docx]

Multimedia Appendix 1

**Variable importance plots**

Figure S1, Figure S2 and Figure S3 show the relative importance of the predictor variables in the LR model and LASSO model, the RP model and RF model and the HC model and NB model, respectively.


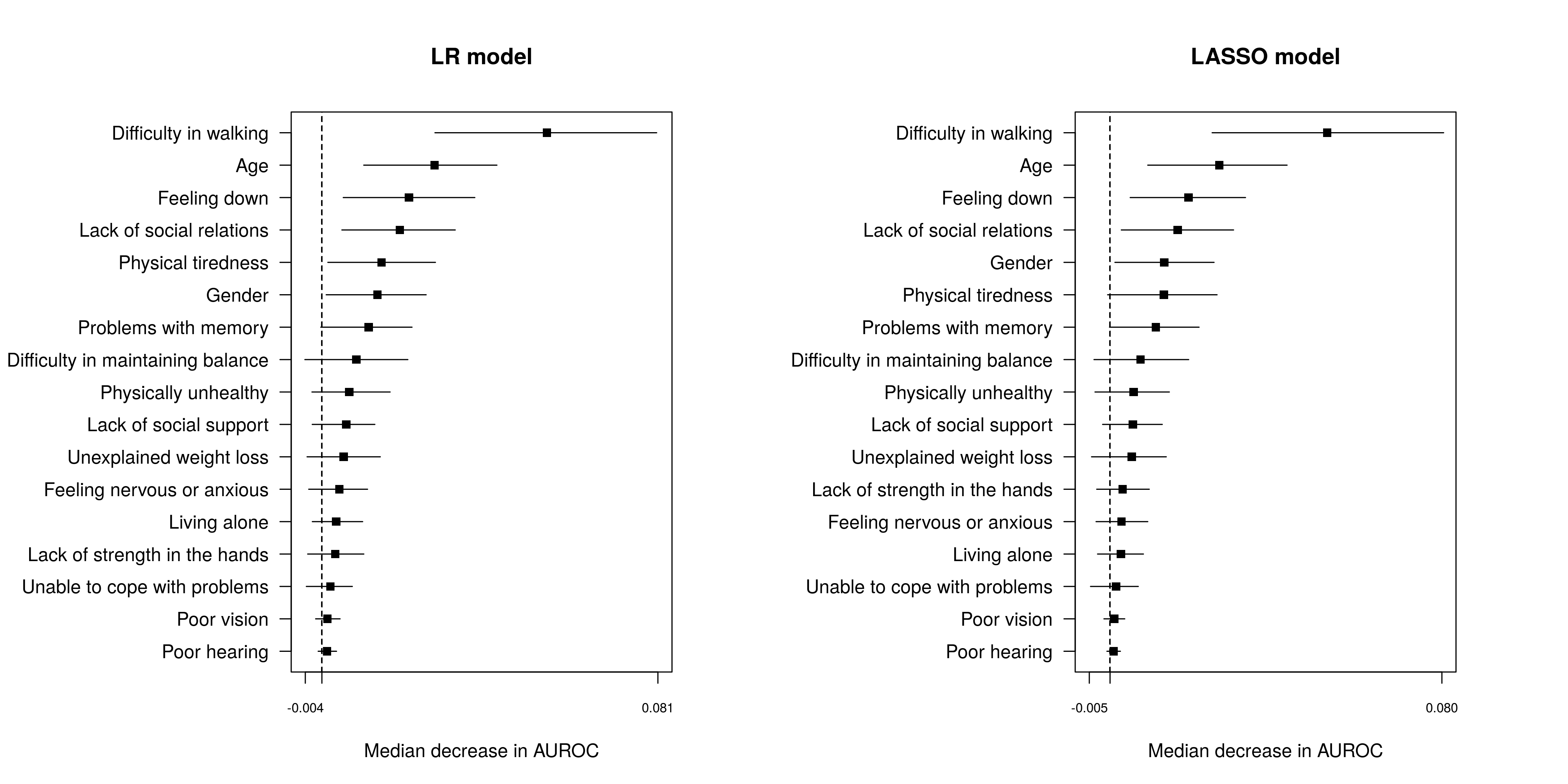


Figure S1: Median decrease in apparent AUC and 95%-CI for LR model and LASSO model


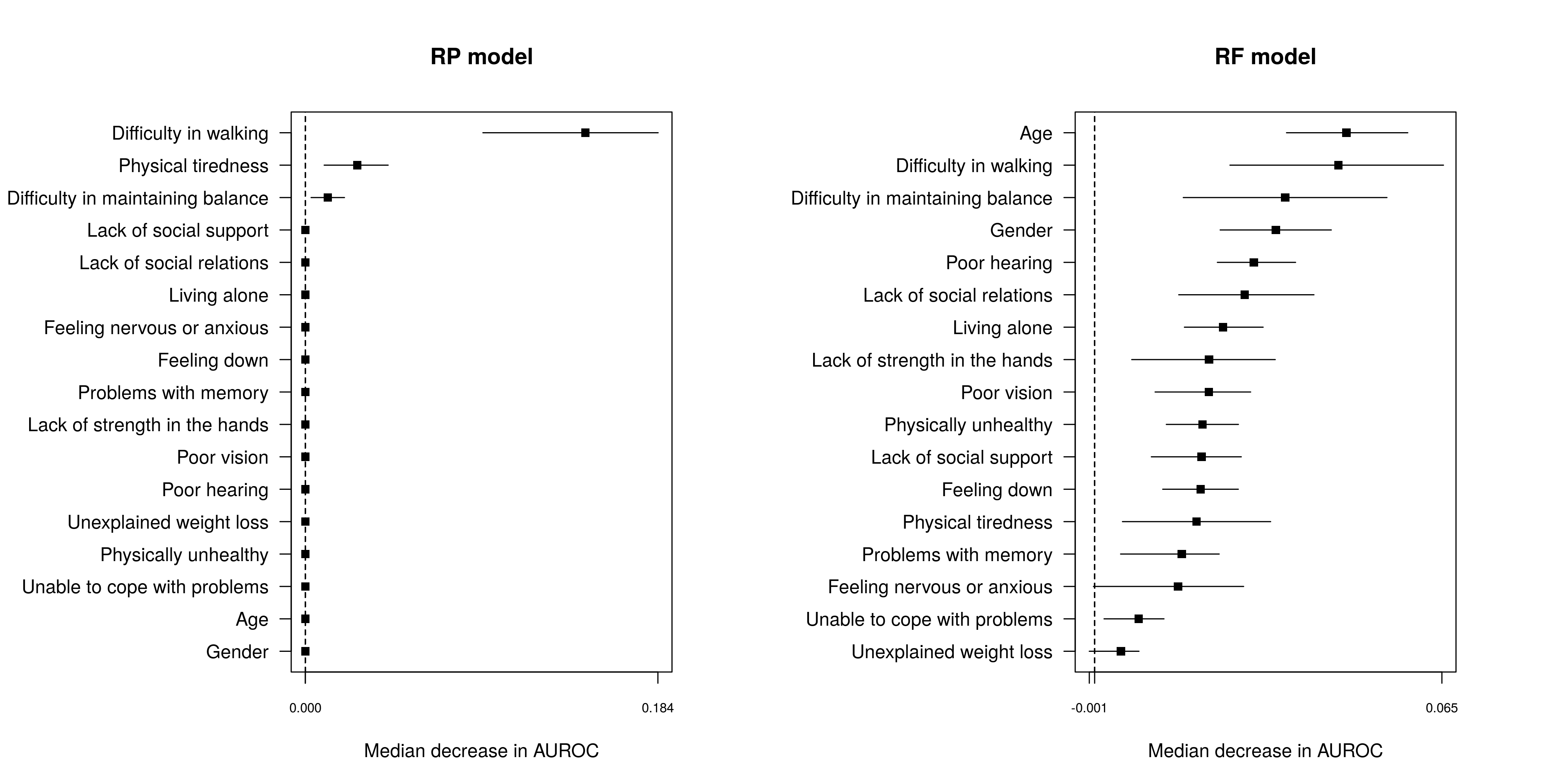


Figure S2: Median decrease in apparent AUC and 95%-CI for RP model and RF model


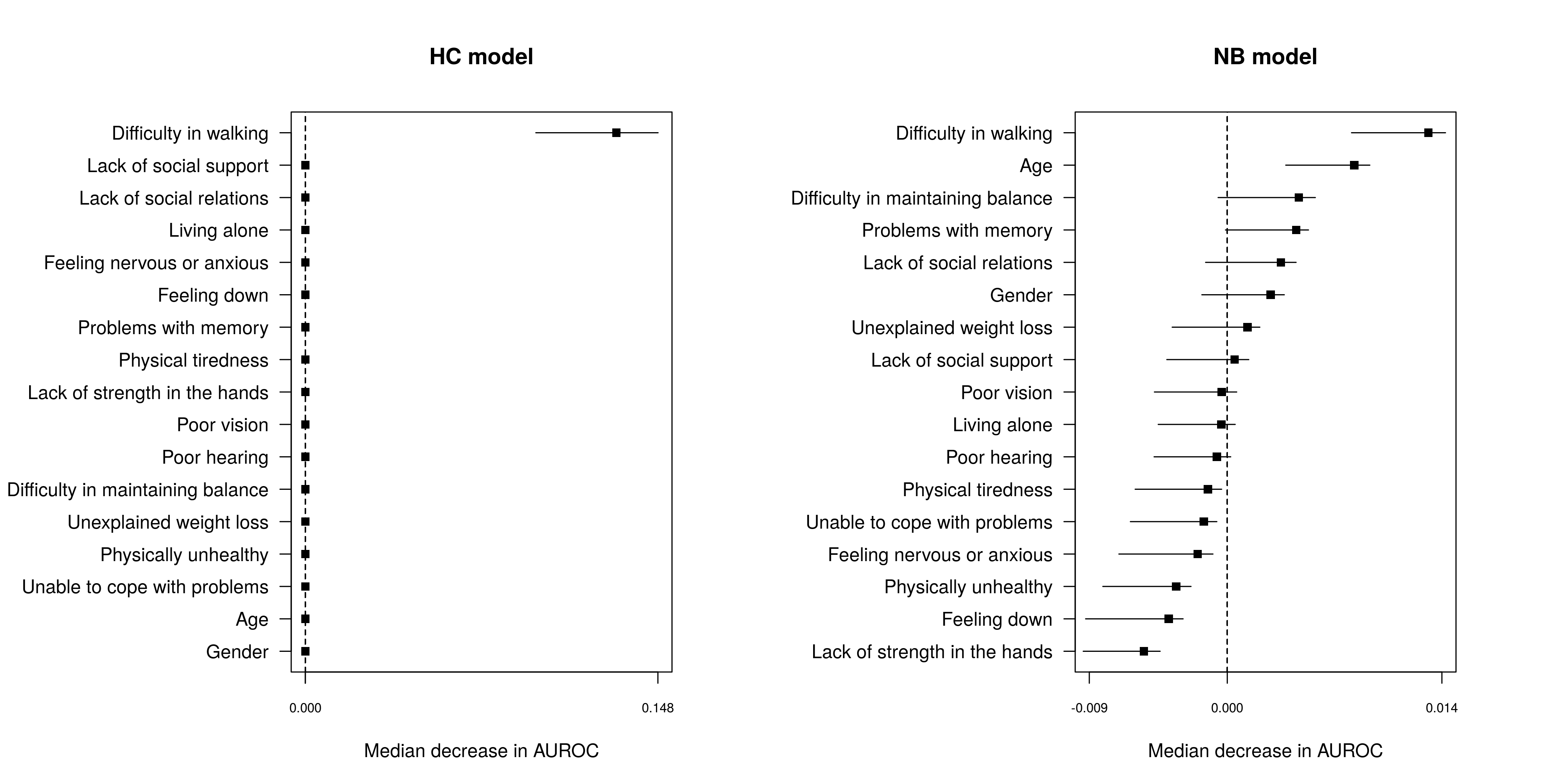


Figure S3: Median decrease in apparent AUC and 95%-CI for HC model and NB model
